# Issues related to the implementation of internship/residency program for qualified medical radiation physicists

**DOI:** 10.4103/0971-6203.48714

**Published:** 2009

**Authors:** S. D. Sharma

**Affiliations:** Radiological Physics and Advisory Division (RPAD), Bhabha Atomic Research Centre (BARC) CTCRS, Anushaktinagar, Mumbai – 400 094, India. E-mail: sdsbarc@gmail.com

Medical radiation physics is the science that primarily deals with the application of ionizing radiation in human health care through radiotherapy, diagnostic radiology, nuclear medicine, and the associated radiation protection and safety. A qualified medical radiation physicist (QMRP) is an individual who is competent to independently practice one or more subfields of medical radiation physics. In a hospital, the QMRP is basically concerned with three different activities, namely clinical service, research and development, and teaching. Services of a QMRP are needed for those medical institutions where radiation generating equipment and sources are used in clinical practice. Availability of a QMRP and a radiological safety officer (RSO) [also called radiation protection officer (RPO)] in medical institutions dealing with radiotherapy is an essential requirement for purposeful and safe usage of ionizing radiations in a cancer management program.[1–4]

Significant advancement has taken place in the technology of radiation delivery and imaging equipment, to make high precision radiotherapy a reality. A number of complex equipments and procedures are used to fulfill the objectives of effective and safe radiotherapy. The use of fully computer controlled ultra-tech equipment (e.g., high-end medical accelerator, CT-Sim) and advanced treatment techniques [e.g., 3-D conformal radiotherapy (3-DCRT), intensity modulated radiotherapy (IMRT), and image-guided radiotherapy (IGRT)] require highly skilled and dedicated physics service. This has resulted in a many-fold increase in the role and involvement of QMRP, in recent radiotherapy practice. While it has long been recognized that the physical aspects of quality assurance in radiotherapy are vital for achieving effective and safe treatment, it has been increasingly acknowledged that a systematic approach is necessary for all the steps within the clinical and technical aspects of a radiotherapy program. A QMRP practicing radiotherapy must have successfully completed academic studies in radiological/ medical physics (typically at a postgraduate level) and sufficient clinical training (about two-three years), majoring in radiotherapy physics.[5] It has therefore been recommended by world bodies and professional organizations that internship/ residency should be incorporated in the curriculum of the medical radiation physics education and training program.[5–10] A mere holder of a university degree in radiological/ medical physics, without the required hospital training, cannot be considered a QMRP. Internship/ residency is now an integral part of the medical physics education and training programs of almost all the countries of Europe, North America, and Australia and a few places in Africa and Asia.[6–11]

In India, at present there are two different pathways to become a QMRP: (1) one year post M. Sc. Diploma in radiological/ medical physics after postgraduate degree in physics from a recognized university, or (2) two years postgraduate degree course in medical physics after graduation in science (majoring in physics) from a recognized university [Table 1]. The radiological/ medical physics education and training programs of the country are basically academic courses that include about three months of clinical observation at a moderately equipped medical institution (see next section), having radiotherapy as one of the major activities. In addition, successful candidates of these courses undergo a centralized certification examination to obtain eligibility for nomination as RSO in medical institution. Because of sparse clinical training, successful candidates of these courses can be considered academically qualified medical radiation physicists, requiring comprehensive clinical training to render the services expected from them. To maintain the content and quality of the radiological/ medical physics courses at par with international standards, it is necessary to incorporate and implement internship/ residency in medical physics education and training programs of the country. Accepting the need for clinical training, the national regulatory authority of India — the Atomic Energy Regulatory Board (AERB) — has incorporated this as an essential requirement for a qualified medical radiation physicist.[2]

**Table 1:** Recognized postgraduate radiological/ medical physics courses in India

*Course Title*	*Institution/ University*	*Course duration (years)*	*Hospital training (months)*	*Intake*
Post M. Sc. Diploma in	RPAD/ BARC [Homi Bhbha National			
Radiological Physics (Dip. R. P.)	Institute (a deemed to be university)], Mumbai	1	2 - 3	30
M. Sc. Medical Physics	Anna University (AU), Chennai	2	2 - 3	25
M. Sc. Medical Physics	Bharathiar University, Coimbatore	2	2 - 3	15
M. Sc. Radiation Physics	Calicut University, Calicut	2	2 - 3	8
M. Sc. Radiation Physics	Manipal University, Manipal	2	> 3	6
P. G. Diploma in Medical Physics	Jadavpur University, Kolkata	1	2 - 3	10
M. Sc. Medical Physics	Punjab University	2	> 3	12
M. Sc. Medical Physics	PSG College of Engineering, Coimbatore (affiliated to AU)	2	2 - 3	15

## Content, duration, and modality of the internship/ residency program

A majority of the radiotherapy (RT) centers in India and other developing countries offer conventional treatments by means of ^60^Co gamma rays from telecobalt units/ megavoltage x-rays (6 MV) from single energy medical linear accelerator. However, moderately equipped centers offer a complete range of treatment options, including IMRT/ IGRT, as both low- and high-end equipments are available at these centers. Specifically, radiation treatment center which is equipped with: (i) a dual photon and multi-electron energy medical linear accelerator containing a multileaf collimator and portal imaging device, (ii) a telecobalt machine, (iii) a radiotherapy simulator, (iv) a high dose rate remote afterloading brachytherapy unit, (v) a 3-D treatment planning system with beam and brachytherapy softwares, (vi) a radiation field analyzer, and (vii) necessary dosimetry, quality assurance and protection instruments can be categorized as a moderately equipped RT center. As far as the requirements and job opportunities in India and other developing countries are concerned, a majority (about 90%) of the QMRP work in RT departments. The course content of the internship/ residency program should therefore be tailored to meet, in general, the requirements of conventional as well as advanced radiotherapy practice, including a few relevant aspects of diagnostic radiology (DR) and nuclear medicine (NM). The radiation safety component should be given due consideration so that the QMRP has sufficient experience to discharge the duty as RSO/ RPO. It is also important that clinical training should include practical details of individual procedures as well as details of how to design treatment approaches that are comprehensive, reproducible, of high quality and safe.[5,7]

Considering the vast content proposed above, the ideal duration for the internship/ residency should be two years. To start with, the internship content could be appropriately designed to include all important components of clinical practice so that it can be completed in one year. In this case, preference should be given to dosimetry, treatment planning, quality control, and radiation safety applied to conventional and 3-D CRT. The clinical and technical aspects of IMRT/ IGRT should be a small component here.

The training modality can be on-job-training (OJT)/ in-service-training (IST) or contracted-intern-training (CIT). The advantage of OJT/ IST is that it may fulfill the financial requirement of the intern, but the institution may appoint interns only when there is a need for an additional QMRP. Hence accepting interns will be an irregular affair for the identified training center, in this mode. On the other hand, the CIT mode may provide financial assistance to the intern and could be a regular program of the identified training center, and hence it is preferable.

Interns/ residents can be assumed as academically trained medical radiation physicists who are capable of providing clinical service under the supervision of a QMRP. In numerical terms of the staffing level, an intern can be assumed as 0.5 QMRP. The numerical staffing level proposed here for an intern/ resident should have the recognition of the national competent authority. This may pave the way for a training center to convince the management to make provision for financial assistance to interns/ residents in lieu of a regular QMRP. Alternatively, either university alone or university and the training center together may make provision for providing financial support to the interns/ residents.

## Training center and the trainer

The training center should be a moderately equipped medical institution with radiotherapy as the main activity. If a well-equipped radiotherapy center does not have a telecobalt machine, two or more centers of the same city can form a group for obtaining recognition as a clinical training site. One of the centers of this group can be designated as the primary training center with the responsibility of arranging the required clinical training for the intern. Similar arrangements can be made in case of non-availability of the DR and NM departments.

The trainer should be a QMRP with at least five years of working experience. The trainer should be a full timer who can be designated as clinical training supervisor (CTS). The CTS should be easily accessible for the intern/ resident for obtaining on the spot guidance. The number of interns/ residents for a training center can be decided on the basis of the available training infrastructure including QMRP.

## Evaluation and certification

A national competent authority should be an evaluating and certifying agency for the interns/ residents. Alternatively, professional bodies/ associations should be empowered by the national competent authority to work as an examining and certifying agency. The professional body should evolve a universal evaluation system that is internationally acceptable.[3,6–8,10] However, in the interim period, the periodic evaluation report of the CTS can be used for issuing the training completion certificate to interns/ residents.

## Harmonization of internship/residency

A harmonized and appropriately structured module should be used for the clinical training of interns/ residents. The International Atomic Energy Agency (IAEA) under the Regional Cooperation Agreement [(RCA), a group of 17 countries from Asia Pacific region)] project “Strengthening of Medical Physics through Education and Training” has evolved a competency based clinical training program for RT physicists. This clinical training program incorporating relevant modifications as per the national needs can be adopted for the training of interns/ residents.
